# Self-rated health, epigenetic ageing, and long-term mortality in older Australians

**DOI:** 10.1007/s11357-024-01211-2

**Published:** 2024-05-25

**Authors:** Danmeng Lily Li, Allison M. Hodge, Melissa C. Southey, Graham G. Giles, Roger L. Milne, Pierre-Antoine Dugué

**Affiliations:** 1 Precision Medicine, School of Clinical Sciences at Monash Health, Monash University, Clayton, VIC Australia; 2 Cancer Epidemiology Division, Cancer Council Victoria, Melbourne, VIC Australia; 3 Centre for Epidemiology and Biostatistics, Melbourne School of Population and Global Health, The University of Melbourne, Parkville, VIC Australia; 4 Department of Clinical Pathology, The University of Melbourne, Parkville, VIC Australia

**Keywords:** Self-rated health, Epigenetic ageing, All-cause mortality, Interaction

## Abstract

Self-rated health (SRH) is a subjective indicator of overall health based on a single questionnaire item. Previous evidence found that it is a strong predictor of mortality, although the underlying mechanism is poorly understood. Epigenetic age is an objective, emerging biomarker of health, estimated using DNA methylation data at hundreds of sites across the genome. This study aimed to assess the overlap and interaction between SRH and epigenetic ageing in predicting mortality risk. We used DNA methylation data from 1059 participants in the Melbourne Collaborative Cohort Study (mean age: 69 years) to calculate three age-adjusted measures of epigenetic ageing: *GrimAge*, *PhenoAge*, and *DunedinPACE*. SRH was assessed using a five-category questionnaire item (“excellent, very good, good, fair, poor”). Cox models were used to assess the associations of SRH, epigenetic ageing, and their interaction, with all-cause mortality over up to 17 years of follow-up (*N*_deaths_ = 345). The association of SRH with mortality per category increase was HR = 1.29; 95%CI: 1.14–1.46. The association was slightly attenuated after adjusting for all three epigenetic ageing measures (HR = 1.25, 95%CI: 1.10–1.41). A strong gradient was observed in the association of *GrimAge* (*P*_*interaction*_ = 0.006) and *DunedinPACE* (*P*_*interaction*_ = 0.002) with mortality across worsening SRH strata. For example, the association between *DunedinPACE* and mortality in participants with “excellent” SRH was HR = 1.02, 95%CI: 0.73–1.43 and for “fair/poor” HR = 1.72, 95%CI: 1.35–2.20. SRH and epigenetic ageing were synergistic risk factors of mortality in our study. These findings suggest that consideration of subjective and objective factors may improve general health assessment, which has implications for the ongoing development of molecular markers of ageing.

**Supplementary Information:**

The online version contains supplementary material available at 10.1007/s11357-024-01211-2.

## Introduction

Self-rated health (SRH) is a widely used subjective summative measure of health referred to as an “active cognitive process” that indicates individuals’ physical and mental health in conjunction with their objective clinical conditions, social characteristics, and even genetic factors [1]. It has been consistently observed that poorer SRH is associated with higher risk of all-cause and cause-specific mortality [2–8]. A substantial body of research has also shown that this association cannot be fully explained by a range of physiological and non-physiological factors that contribute to health [1, 4, 9], which highlights that, despite being simple and subjective, SRH provides a valuable assessment of health.

SRH is associated with many biomarkers of ageing. In a study that included over 15,000 adults sampled from three cohorts, SRH was associated with 57 out of 150 biomarkers that are closely related to physiological regulation [10]. Poor SRH has consistently been found to be associated with low-grade inflammation and elevated levels of inflammatory biomarkers such as interleukin-6 and C-reactive protein (CRP) [11–14] which are important indicators of biological ageing [15–17]. Many studies have examined the relative value of SRH and objective markers of health in predicting mortality. A study based on nearly 500,000 participants in the UK Biobank assessed the associations of 655 demographic, lifestyle, and health markers with 5-year all-cause mortality and found that SRH was more strongly predictive of mortality than the majority of more objective markers of health such as walking pace, illnesses and disability [18]. Another study of 8138 participants in the Northern Ireland Cohort for the Longitudinal Study of Ageing found that the risk of mortality associated with poor SRH was higher than with limiting long-term illnesses (any long-term illnesses or disability and whether these conditions limited activities), but lower than with a modified Charlson Index (calculated based on 16 selected chronic conditions) [4]. Some studies also explored how SRH might interact with objective health indicators in their association with mortality [7, 19], and one found that the association of SRH with mortality was stronger in participants with type II diabetes [19].

DNA methylation (DNAm)-based markers of ageing, “epigenetic ageing”, are well-established and powerful measures of biological age [20, 21]. A previous review that compared six biological age predictors: epigenetic age, telomere length, transcriptomic, proteomic and metabolomics-based predictors, and composite biomarker predictors, concluded that epigenetic age is currently the most valid and predictive measure of biological age [22], and epigenetic ageing markers have been considerably improved since the review was published [23–26]. Therefore, we hypothesised that with epigenetic ageing being such an excellent estimator of biological age, it might contribute to explain the mechanisms by which SRH relates to poor health at the molecular level.

Limited research has investigated the link between SRH and epigenetic ageing. *DunedinPACE*, a third-generation epigenetic clock, was found to be correlated with SRH [25]. In another cohort study, SRH was reported to mediate the association between subjective age (how old individuals perceived themselves relative to their chronological age) and *PhenoAge* [27]. Although the respective individual associations of SRH and epigenetic ageing with mortality have been studied extensively [2, 4–6, 28, 29], there is a lack of evidence on i) whether epigenetic ageing could provide an explanation for the SRH/mortality association; ii) whether epigenetic ageing measures, developed using clinical markers, encompass subjective aspects of the ageing process; and iii) whether these subjective and objective markers could have a synergistic effect on mortality prediction. Therefore, we investigated the overlap and interaction between SRH and epigenetic ageing to help explain the underlying mechanism of SRH in predicting mortality and inform precision prevention of mortality and morbidity.

This study aimed to assess in a large sample of middle-aged and older Australians: 1) the extent to which epigenetic ageing captures the association between SRH and mortality, and 2) the joint effect of epigenetic ageing and SRH on mortality.

## Methods

### Study participants

The Melbourne Collaborative Cohort Study (MCCS) is a prospective cohort study of 41,513 middle-aged to older Australians (59% females) of white European origin who were aged between 40 and 69 (99% of them) at recruitment between 1990 and 1994 (baseline) [30]. A face-to-face follow-up (follow-up 2) was carried out between 2003 and 2007 and data on lifestyle, health status, physical measurements and blood samples were collected [30].

The present study used follow-up 2 data of a subset of 1100 controls from six cancer case–control studies nested in the MCCS. Genome-wide DNA methylation was measured in participants who had provided blood samples (stored as dried blood spot on a Guthrie card) at follow-up using the Illumina HumanMethylation450K BeadChip array [30]. Details of DNA extraction and processing and quality control of methylation data have been described elsewhere [31–33]. After excluding participants who failed DNAm quality control (*n* = 12, 1%) and those with missing data for SRH (*n* = 29, 3%), 1059 participants were included in the analysis.

Participants were passively followed from the time they attended the follow-up interview to 31st October, 2019, and deaths were identified by annual record linkage to the Victorian Registry of Births, Deaths and Marriages [30].

The MCCS was approved by the Human Research Ethics Committee of the Cancer Council Victoria, Melbourne, VIC, Australia, and informed consent was provided by all participants according to the Declaration of Helsinki.

### Self-rated health

SRH was one item extracted from the 12-Item Short Form Survey [1, 34] used at follow-up 2 and collected by asking participants: “In general, would you say your health is …?” with five response options provided: excellent, very good, good, fair, and poor. Since only 20 participants reported “poor” health the “poor” and “fair” categories were merged into one “fair/poor” category. SRH was also used as a pseudo-continuous variable scored from 0 to 3, with 0 representing “excellent” health.

### Epigenetic ageing

Three epigenetic ageing measures that are widely used and most strongly associated with mortality were considered in this study: *GrimAge*, *PhenoAge*, and *DunedinPACE*. *GrimAge* was developed with chronological age, sex, and DNAm predictors of smoking pack-years and seven plasma proteins based on 1030 CpGs [24]. *PhenoAge* incorporated 513 CpGs selected based on chronological age and nine clinical markers predictive of mortality [23]. *DunedinPACE* measures the rate of ageing with 173 CpGs based on the Pace of Ageing calculated from changes in 19 biomarkers [25]. In the MCCS, *GrimAge* and *PhenoAge* were calculated using the Horvath Lab’s web tool (http://dnamage.genetics.ucla.edu/new), and *DunedinPACE* using the R code provided in the original publication [25, 35]. All three measures were regressed on chronological age and the residuals were used as the age-adjusted measures of epigenetic ageing in all analyses. Since these measures are expressed on a different scale, we standardised them to a mean of 0 and standard deviation of 1 in the survival analyses.

### Confounders

We considered age, sex, smoking status (never/former/current smokers), and country of birth (Australia/New Zealand/other, UK/Malta, Greece, and Italy) as major confounding factors. Physical measurements on waist circumference, weight, height, blood pressure, and resting heart rate were carried out by trained personnel and the average values from 2–3 measurements were calculated [30]. BMI (kg/m^2^) was calculated using weight and height data. Blood glucose concentration (mmol/L) was measured with a glucometer (95% fasted). The low-density (LDL) and high-density lipoprotein (HDL) cholesterol concentrations were also measured [30]. A fatigue score (soma-6) was created by summing the SOMA-6 part of the 12-item Somatic and Psychological Health Report questionnaire [36] where one point was assigned to each item (ranges 0–6, with higher score representing severer fatigue).

### Statistical analysis

Pearson correlation coefficients of SRH with epigenetic ageing and between different epigenetic ageing measures (all age-adjusted) were calculated. Since *GrimAge* was developed based on sex and a DNAm-based marker of smoking pack-years [24], we expected that it would capture the effects of smoking and sex on mortality risk; to validate the logic of our study, we compared Cox models including these factors with and without adjustment for *GrimAge*.

A series of Cox proportional hazard models with time since blood draw as the time-scale were applied to assess the association between SRH and mortality. Four models were considered: 1) adjusted for age at blood draw, sex, and country of birth; 2) additionally adjusted for each individual epigenetic ageing measure; 3) adjusting for all three epigenetic ageing measures; 4) adding an interaction term between SRH and epigenetic ageing to Model 2. Likelihood ratio tests comparing Model 4 to Model 2 were applied to test for potential interactions between SRH and epigenetic age. The concordance statistic (c-index) was calculated for all models to evaluate the prediction of mortality obtained for different combinations of subjective and objective health indicators.

### Sensitivity analysis

To further assess the independence of the association for SRH with mortality, we added more covariates to Model 2, including smoking status, waist circumference, height, BMI, blood glucose concentration, HDL and LDL cholesterol concentrations, systolic blood pressure, resting heart rate, and soma-6 score.

All statistical analyses were conducted with R version 4.2.2 (Vienna, Austria, 2022) and all *P*-values were two-sided.

## Results

### Sample characteristics

The average age of the 1059 participants at follow-up 2 was 68.7 years (SD = 8.1) and 345 (33%) died over a mean follow-up of 13.0 years (range: 0.3–17.2 years), Table 1. Participants reporting poorer SRH were older than those reporting better SRH (e.g. mean age for “poor” health: 74.1 years; for “excellent” health: 66.6 years). SRH was positively correlated with epigenetic ageing (Figure S1), and the correlation coefficients were greater than that with chronological age (*r* = 0.15), ranging between *r* = 0.16 for *PhenoAge* and *r* = 0.23 for *DunedinPACE*, Table S1. There was moderate correlation between epigenetic ageing measures with the strongest correlation between *GrimAge* and *DunedinPACE* (*r* = 0.61).

**Table 1:** Characteristics of sample participants from the Melbourne Collaborative Cohort Study, Melbourne, Australia, (*n* = 1059, *n*_deaths_ = 345)

Variables	All		Self-rated health							
			Excellent (*n* = 173)		Very good (*n* = 417)		Good (*n* = 335)		Fair/Poor (*n* = 134)	
Age (years), mean (SD)	68.7	(8.1)	66.6	(7.8)	68.3	(8.0)	69.3	(8.1)	71.1	(8.1)
Gender, N (%)										
Female	337	(31.8)	68	(20.2)	135	(40.1)	96	(28.5)	38	(11.3)
Male	722	(68.2)	105	(14.5)	282	(39.1)	239	(33.1)	96	(13.3)
Country of birth, N (%)										
Aust/NZ/Other	823	(77.7)	142	(17.3)	354	(43.0)	237	(28.8)	90	(10.9)
Greece	37	(3.5)	3	(8.1)	5	(13.5)	14	(37.8)	15	(40.5)
Italy	79	(7.5)	7	(8.9)	13	(16.5)	43	(54.4)	16	(20.3)
UK/Malta	120	(11.3)	21	(17.5)	45	(37.5)	41	(34.2)	13	(10.8)
Soma-6 score, median (IQR)	6	(6, 8)	6	(6, 7)	6	(6, 7)	7	(6, 9)	9	(7, 11)
Smoking status, N (%)										
Never smokers	539	(50.9)	102	(18.9)	229	(42.5)	149	(27.6)	59	(11.0)
Current smokers	60	(5.7)	6	(10.0)	15	(25.0)	25	(41.7)	14	(23.3)
Former smokers	460	(43.4)	65	(14.1)	173	(37.6)	161	(35.0)	61	(13.3)
Waist circumference (cm), mean (SD)	94.3	(12.0)	90.3	(11.1)	93.6	(11.7)	96.2	(12.4)	97.2	(11.1)
Blood glucose level (mmol/L), mean (SD)	5.6	(1.1)	5.4	(1.0)	5.6	(1.1)	5.6	(1.0)	5.9	(1.4)
LDL cholesterol level (mmol/L), mean (SD)	2.9	(0.8)	3.2	(0.8)	2.9	(0.9)	2.8	(0.8)	2.7	(0.8)
HDL cholesterol level (mmol/L), mean (SD)	1.5	(0.4)	1.6	(0.4)	1.5	(0.4)	1.5	(0.4)	1.5	(0.4)
Systolic blood pressure (mmHg), mean (SD)	137.3	(17.9)	135.9	(17.1)	137.1	(17.3)	138.4	(18.0)	137.7	(21.1)
Resting heart rate (beats/min), mean (SD)	64.5	(10.2)	63.8	(10.3)	64.2	(9.8)	64.5	(10.2)	66.5	(11.3)
Age-adjusted epigenetic age, mean (SD)										
*GrimAge* (years)	0	(4.2)	-1.3	(3.8)	-0.4	(3.8)	0.7	(4.4)	1.2	(4.5)
*PhenoAge* (years)	0	(6.9)	-1.7	(6.7)	-0.5	(7.0)	0.8	(6.8)	1.9	(6.7)
*DunedinPACE*	0	(0.1)	-0.04	(0.1)	-0.01	(0.1)	0.02	(0.1)	0.05	(0.1)
Mortality, N (%)										
Alive	714	(67.4)	131	(18.4)	298	(41.7)	218	(30.5)	67	(9.4)
Dead	345	(32.6)	42	(12.2)	119	(34.5)	117	(33.9)	67	(19.4)
Follow-up time (years), mean (SD)	13.0	(3.5)	14.1	(2.7)	13.4	(3.2)	12.8	(3.5)	11.1	(4.5)

^*^SD standard deviation; *IQR* interquartile range; *HDL* high-density lipoprotein; *LDL* low-density lipoprotein

### Smoking, sex, and mortality

Compared to never smokers, current smokers had strongly elevated mortality risk (HR = 2.3, 95%CI: 1.4–3.7; Table S2) as did former smokers (HR = 1.3; 95%CI: 1.1–1.7). After adjusting for *GrimAge*, these associations were very close to null: current smokers: HR = 1.1, 95%CI: 0.7–2.0, former smokers: HR = 1.0, 95%CI: 0.8–1.3, whereas the association for *GrimAge* remained strong (per SD: HR = 1.4; *P* = 5 × 10^–8^). Similarly, the mortality risk for males compared to females attenuated from 1.4 (95%CI: 1.1–1.8) to 1.1 (95%CI: 0.9–1.5) after adjusting for *GrimAge*. There was also some but less significant reduction of the HRs for smoking status and sex after adjusting for *DunedinPACE* (e.g. for current smokers compared to never smokers: HR = 1.7, 95%CI: 1.0–2.8). Conversely, these associations remained virtually unchanged after adjustment for SRH and *PhenoAge* (Table S2).

### SRH and mortality

Participants reporting “fair/poor” health had substantially higher mortality than those reporting “excellent” health (HR = 2.02; 95%CI: 1.36–2.99; Table 2), whereas those reporting “very good” health had a similar mortality risk (HR = 1.01; 95%CI: 0.71–1.43). There was small attenuation of the HRs after adjusting for epigenetic ageing. For example, after adjusting for *GrimAge*, the HR for “fair/poor” health compared to “excellent” health attenuated to 1.85 (95%CI: 1.25–2.75).

**Table 2:** Cox proportional hazard models for categorical self-rated health (SRH) and all-cause mortality (*n* = 1059, *n*_deaths_ = 345)

Models		SRH									
		Excellent	Very good			Good			Fair/Poor		
			HR	95% CI	*P*-value	HR	95% CI	*P*-value	HR	95% CI	*P*-value
Model 1^a^	Age, sex, and country of birth	Ref. level	1.01	0.71–1.43	0.97	1.28	0.90–1.84	0.17	2.02	1.36–2.99	4 × 10^–4^
Model 2^b^	Adjusted for *GrimAge*		0.97	0.68–1.38	0.87	1.20	0.84–1.72	0.31	1.85	1.25–2.75	0.002
	Adjusted for *PhenoAge*		0.97	0.68–1.38	0.85	1.21	0.84–1.73	0.31	1.86	1.25–2.76	0.002
	Adjusted for *DunedinPACE*		0.94	0.66–1.34	0.73	1.18	0.83–1.69	0.36	1.80	1.21–2.68	0.003
Model 3^c^	Adjusted for all 3 measures		0.95	0.67–1.35	0.78	1.17	0.82–1.68	0.39	1.79	1.21–2.66	0.004

^a^Model 1 adjusted for age, sex, and country of birth. ^b^Model 2 additionally adjusted for epigenetic age. ^c^Model 3 adjusted for all three epigenetic ageing measures

Epigenetic ageing measures were standardised to a mean of 0 and standard deviation of 1

When SRH was analysed as a pseudo-continuous variable, a unit increase in SRH was associated with a 1.29-fold (95%CI: 1.14–1.46, *P* = 4 × 10^–5^; Table 3) increase in mortality risk, whereas the HR per SD increase of *GrimAge* was HR = 1.45 (*P* = 5 × 10^–11^), which was greater than for *DunedinPACE* (per SD, HR = 1.34;* P* = 9 × 10^–8^) and *PhenoAge* (per SD, HR = 1.20;* P* = 2 × 10^–4^). The HR for SRH decreased to 1.25 (95%CI: 1.11–1.42) after adjusting for *GrimAge* or *DunedinPACE* and to 1.26 (95%CI: 1.11–1.43) after adjusting for *PhenoAge*; it was not further attenuated after adjustment for all three epigenetic ageing measures: HR = 1.25 (95%CI: 1.10–1.41). Similarly, there was only negligible attenuation of the HRs for epigenetic ageing after adjusting for SRH (e.g. *GrimAge*: HR = 1.43, 95%CI: 1.28–1.60).

**Table 3:** Cox proportional hazard models for pseudo-continuous self-rated health and all-cause mortality (*n* = 1059, *n*_deaths_ = 345)

Models		SRH (per unit)			*GrimAge* (per SD)			*PhenoAge* (per SD)			*DunedinPACE* (per SD)		
		HR	95% CI	*P*-value	HR	95% CI	*P*-value	HR	95% CI	*P*-value	HR	95% CI	*P*-value
Model 1^a^	Age, sex, and country of birth	1.29	1.14–1.46	4 × 10^–5^	1.45	1.30–1.62	5 × 10^–11^	1.20	1.09–1.32	2 × 10^–4^	1.34	1.20–1.49	9 × 10^–8^
Model 2^b^	Adjusted for *GrimAge* (resp. SRH)	1.25	1.11–1.42	3 × 10^–4^	1.43	1.28–1.60	4 × 10^–10^						
	Adjusted for *PhenoAge* (resp. SRH)	1.26	1.11–1.43	3 × 10^–4^				1.18	1.06–1.30	0.001			
	Adjusted for *DunedinPACE* (resp. SRH)	1.25	1.11–1.42	3 × 10^–4^							1.31	1.18–1.46	8 × 10^–7^
Model 3^c^	Adjusted for all 3 measures	1.25	1.10–1.41	5 × 10^–4^	1.33	1.16–1.53	4 × 10^–5^	1.01	0.91–1.13	0.81	1.12	0.99–1.28	0.08

^a^Model 1 adjusted for age, sex, and country of birth. ^b^Model 2 additionally adjusted for epigenetic age. ^c^Model 3 adjusted for all three epigenetic ageing measures and SRH score

Epigenetic ageing measures were standardised to a mean of 0 and standard deviation of 1

### Interaction between SRH and epigenetic ageing

**Fig. 1:**
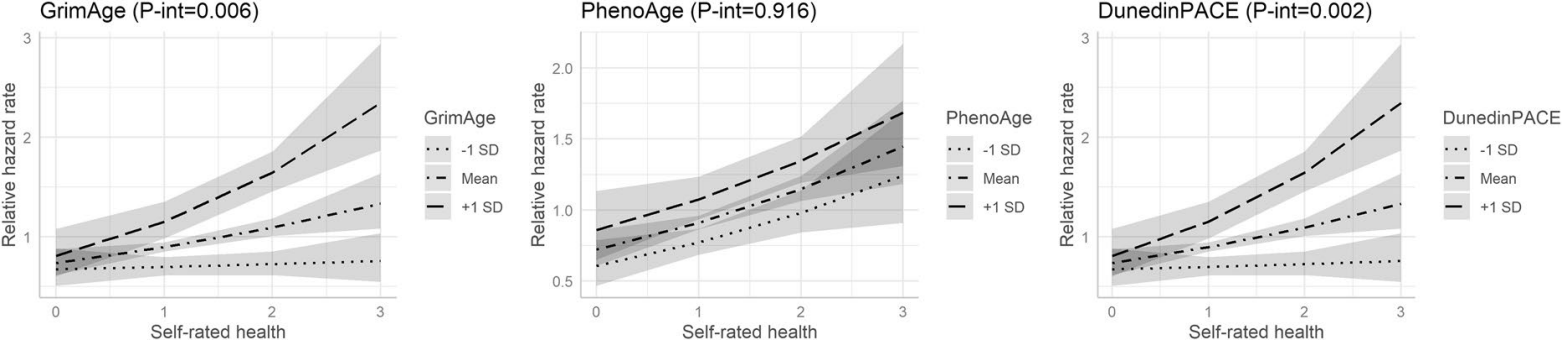
Interaction between SRH and epigenetic ageing measures in their associations with all-cause mortality (*n* = 1059, *n*_deaths_ = 345). *Models adjusted for age, sex, country of birth, SRH (continuous), epigenetic ageing, and an interaction term between SRH and epigenetic ageing. All epigenetic ageing measures were standardised to a mean of 0 and standard deviation of 1

**Fig. 2:**
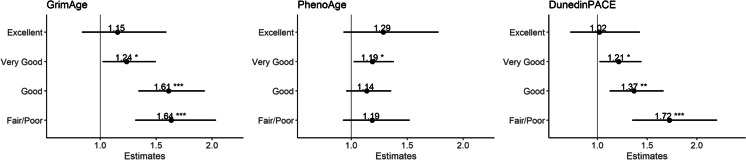
Associations of epigenetic ageing with all-cause mortality across self-rated health strata (*n* = 1059, *n*_deaths_ = 345). *Models adjusted for age, sex, country of birth, SRH (categorical), epigenetic ageing, and an interaction term between SRH and epigenetic ageing. All epigenetic ageing measures were standardised to a mean of 0 and standard deviation of 1

We observed strong evidence of interactions of SRH with *GrimAge* (*P* = 0.006) and *DunedinPACE* (*P* = 0.002), but not with *PhenoAge* (*P* = 0.92), with a stronger association of SRH with mortality in participants with higher *GrimAge* or *DunedinPACE* (Fig. 1). This is further illustrated in Fig. 2, showing a strong gradient across SRH strata for the association of *GrimAge* (or *DunedinPACE*) with mortality, which was small in participants reporting “excellent” SRH (*GrimAge*: HR = 1.15, 95%CI: 0.84–1.59; *DunedinPACE*: HR = 1.02, 95%CI: 0.73–1.43), and very large in those reporting “fair/poor” SRH (*GrimAge*: HR = 1.64, 95%CI: 1.31–2.04; *DunedinPACE*: HR = 1.72, 95%CI: 1.35–2.20).

### Sensitivity analysis

Very similar results were observed in the association between SRH and mortality after adjustment of a large number of health-related lifestyle variables and biomarkers (based on 802 participants, adjusting for *GrimAge*: HR = 1.28; 95%CI: 1.09–1.50; Table S3). There was also no notable change to the associations of epigenetic ageing measures with mortality.

### Contribution to prediction

In univariate models, the c-index was 0.589 for SRH, 0.590 for *GrimAge*, 0.552 for *PhenoAge*, 0.574 for *DunedinPACE*, which was substantially lower than Model 0 (age, sex, and country of birth, c = 0.748), Table S4. Adding SRH to Model 0 (Model 1) increased the c-index to 0.756. This was slightly lower than for *GrimAge* (*c* = 0.763) and *DunedinPACE* (*c* = 0.760), but higher than for *PhenoAge* (*c* = 0.754). Adding epigenetic ageing to Model 1 further increased the c-index (e.g. *GrimAge*: *c* = 0.768) with minimal increase after including all three epigenetic clocks (*c* = 0.769). Models using continuous or categorical SRH had similar c-indices. Some further increase in the c-index was observed after including an interaction term between SRH score and epigenetic ageing (e.g. *GrimAge*: *c* = 0.771). In the sensitivity analysis, inclusion of more covariates in the model also increased the c-index (to 0.782 for *GrimAge* and to 0.777 for *PhenoAge* and *DunedinPACE*).

## Discussion

This study found that the association of SRH with long-term all-cause mortality was largely not explained by a group of objective markers of biological ageing (epigenetic age) in a large sample of middle-aged and older Australians. Both subjective and objective health assessments showed strong associations with mortality. *GrimAge* appeared to be the best mortality predictor among the four, followed by *DunedinPACE*, while SRH and *PhenoAge* showed similar results in mortality prediction. Combining SRH and epigenetic age improved mortality prediction of the models. *GrimAge* and *DunedinPACE* strongly interacted with SRH, with risk of death being much stronger in participants with higher *GrimAge* or *DunedinPACE* and poorer SRH. No interaction was observed for *PhenoAge*, which is an epigenetic ageing measure less strongly predictive of mortality. Lifestyle-related variables and clinical markers did not appear to explain any of the associations of SRH and epigenetic ageing with mortality.

Our findings on the association between SRH and mortality are consistent with previous studies, where only part of the SRH/mortality association could be explained by a wide range of indicators of physical function, biomarkers of ageing and inflammation, medical and psychological conditions, and lifestyle and psycho-social factors [2–7, 9, 10, 37, 38]. For example, in the study by Kananen et al., the relative risk of mortality for “poor” SRH compared to “good” SRH attenuated from 2.6 to 2.4 after adjusting for the number of chronic diseases, and to 2.0 after further including ten biomarkers that were strongly associated with SRH [10]. A study based on the Chinese Longitudinal Healthy Longevity Survey observed a relative risk attenuation of 15% after adjusting for major health risk factors including chronic diseases and cognitive impairment [38]. Although investigating the interactions between SRH and objective health indicators may help identify population subgroups more suitable to receive health interventions according to their health status, to our knowledge very few studies have examined such interactions [7, 19]. Wuorela et al. found no interaction between SRH and a frailty index [7], whereas Dankner et al. observed that the association between SRH and mortality was stronger in participants with type II diabetes than their normoglycemic counterparts [19].

Similarly, epigenetic ageing was found to be largely independent of other biological age estimators. In 1314, Scottish individuals aged 70–90 years [39] the first-generation epigenetic clocks and telomere length were independently associated with mortality. Using MCCS data, we previously found that inflammaging (increased concentrations of age-related inflammation markers) only explained a small amount of the association between epigenetic ageing and mortality [40]. Another study assessed the longitudinal association between nine biological ageing measures (including four epigenetic clocks) and mortality and reported that *GrimAge* and *HorvathAge*, but not *PhenoAge* and *HannumAge*, were independently associated with mortality [41].

The variation between epigenetic ageing measures also aligned with existing evidence. These measures were developed to estimate different aspects of ageing: *PhenoAge* was developed to predict phenotypic age based on ten clinical markers [23]; *GrimAge* was developed based on eight DNAm-based surrogate markers to predict mortality [24], while *DunedinPACE* used DNAm-based surrogated markers of rate of changes in 19 selected biomarkers in four waves of follow-up over 20 years to estimate the rate of ageing [25]. Such variation can be seen in previous studies. For example, *DunedinPACE* had the strongest association with obesity compared to *PhenoAge* and *GrimAge* since it incorporates more obesity-related markers of ageing [42], *PhenoAge* and *GrimAge* showed slightly different patterns of association with risk of various cancers, *GrimAge* being more strongly associated with lung and urothelial cancer risk, which are mainly smoking-related [43]. A randomized controlled trial found that caloric restriction caused reduction only in *DunedinPACE*, but not in *GrimAge* or *PhenoAge* [44].

Despite intense exploration of both SRH and epigenetic ageing in previous studies, to our knowledge, this is the first time these two variables have been considered jointly and a strong interaction was found between them. Epigenetic ageing measures, which have been developed based on clinical markers to predict mortality, appeared to be better predictors of mortality in those reporting fair/poor health, and were less effective in participants reporting to be very healthy. Our study therefore highlights that SRH, as a subjective measure of health which plays an important role in mortality prediction regardless of many objective health indicators, might have important implications for the interpretation and ongoing development of biological age estimators. Future studies could also explore the associations between other epigenetic markers and SRH, which could be helpful in developing potential epigenetic measures that capture subjective aspects of ageing.

This study has some limitations. Although our sample was reasonably large, our findings should be replicated and extended in additional and larger studies. The MCCS participants were healthier than the general population [45], which may not have caused major bias because selection bias is usually considered to be small and a previous study reported that excluding participants who were too sick to respond to SRH questions had little impact on the effect estimates of the SRH/mortality association [38]. A shared limitation of our study and others is that SRH was collected at only one time point. Since SRH is expected to change with time [1], SRH information collected at additional time points might capture its association with mortality more accurately. In our study, the participants were followed for an average of 13 years and both SRH and epigenetic ageing measures were less predictive of long-term than short-term mortality, but the results were materially unchanged when follow-up time was restricted to five or ten years (not shown).

Evidence from our study and others suggests that the mechanisms underlying the association between SRH and mortality are not fully explained by objective factors including diseases, biomarkers, lifestyle, and psycho-social factors usually collected in large epidemiological studies. Although the epigenetic biomarkers of ageing we assessed use information from many DNAm sites (173 CpGs for *DunedinPACE* to 1030 GpGs for *GrimAge*), were designed to capture the collective effect of a large number of health-related variables and biomarkers of physiological functions, and have been widely reported to be excellent markers of ageing, they also accounted for a small proportion of the SRH/mortality association. This conclusion was further supported by our validation test showing that *GrimAge* almost entirely captured the effect of smoking and sex on mortality [24]. Future studies could consider other biological, clinical, and psycho-social variables that may explain why individuals reporting their health to be poor die younger.

In conclusion, SRH and epigenetic ageing were correlated with each other, both strongly associated with mortality, and showed a strong interaction whereby the associations of *GrimAge* and *DunedinPACE* with mortality was close to null in participants reporting excellent health and very large in those reporting poor health. These findings highlight that both subjective and objective markers of ageing are important to comprehensively evaluate an individual’s health and risk of mortality. This has implications for the ongoing development of molecular markers of ageing. Additional research efforts are required to uncover the mechanisms underlying the association between SRH and mortality and identify potential molecular markers underlying poor self-rated health.

## Supplementary Information

Below is the link to the electronic supplementary material.

Supplementary file1 (DOCX 240 KB)

## Data Availability

Due to ethical constraints related to the consent of participants, we cannot share the full deidentified dataset. For most participants included in this study, the data are publicly available under controlled-access at dbGaP (#phs003213.v1.p1, for which more details can be found at https://www.ncbi.nlm.nih.gov/projects/gap/cgi-bin/study.cgi?study_id=phs003213.v1.p1).
