# Supplementary material for: Self-rated health, epigenetic ageing, and long-term mortality in older Australians
Source: GeroScience. 2024 May 25;46(6):5505–15. doi: 10.1007/s11357-024-01211-2 (PMC11493901; doi:10.1007/s11357-024-01211-2)
Supplement: Supplementary file 1 — Supplementary file1 (DOCX 240 KB) [file 11357_2024_1211_MOESM1_ESM.docx]

Figure S1: Box and Whisker plots for chronological age and epigenetic ageing across self-rated health (SRH) strata.


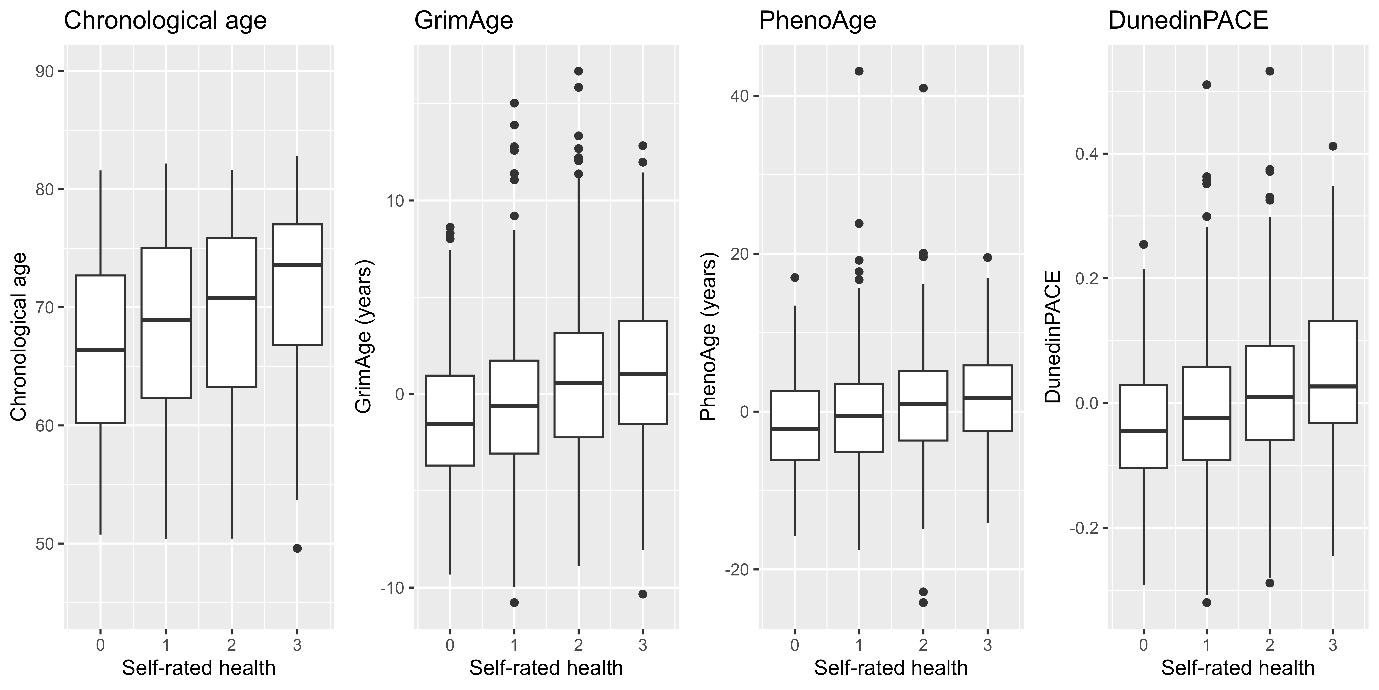


| Variables | SRH | *GrimAge* | *PhenoAge* | *DunedinPACE* | Chronological age |
| --- | --- | --- | --- | --- | --- |
| SRH | 1.00 |  |  |  |  |
| *GrimAge* | 0.19 | 1.00 |  |  |  |
| *PhenoAge* | 0.16 | 0.43 | 1.00 |  |  |
| *DunedinPACE* | 0.23 | 0.61 | 0.40 | 1.00 |  |
| Chronological age | 0.15 | -0.01 | 0.00 | -0.01 | 1.00 |

Table S1: Correlation matrix of SRH and epigenetic ageing measures.

*SRH = self-rated health. All epigenetic ageing measures are age-adjusted.

Table S2: The association between smoking and mortality (*n*=1059, *n*_deaths_=345).

| **Characteristic** | | **Model 1^a^** | | | **Model 2^b^** | | | | | | | | | **Model 3^c^** | | |
| --- | --- | --- | --- | --- | --- | --- | --- | --- | --- | --- | --- | --- | --- | --- | --- | --- |
|  |  |  |  |  | **GrimAge** | | | **PhenoAge** | | | **DunedinPACE** | | |  |  |  |
|  |  | **HR** | **95% CI** | ***P*-value** | **HR** | **95% CI** | ***P*-value** | **HR** | **95% CI** | ***P*-value** | **HR** | **95% CI** | ***P*-value** | **HR** | **95% CI** | ***P*-value** |
| Smoking status | Never smokers | Reference level | | | | | | | | | | | | | | |
|  | Current smokers | 2.26 | 1.39, 3.68 | 0.001 | 1.13 | 0.65, 1.95 | 0.67 | 2.07 | 1.27, 3.38 | 0.003 | 1.69 | 1.02, 2.80 | 0.040 | 2.13 | 1.31, 3.47 | 0.002 |
|  | Former smokers | 1.33 | 1.06, 1.67 | 0.013 | 1.01 | 0.78, 1.29 | 0.97 | 1.26 | 1.00, 1.58 | 0.053 | 1.24 | 0.99, 1.57 | 0.064 | 1.30 | 1.04, 1.64 | 0.022 |
| Sex | Female | Reference level | | | | | | | | | | | | | | |
|  | Male | 1.40 | 1.08, 1.80 | 0.01 | 1.13 | 0.87, 1.47 | 0.37 | 1.36 | 1.06, 1.76 | 0.017 | 1.27 | 0.98, 1.64 | 0.068 | 1.38 | 1.07, 1.78 | 0.012 |

^a^Model 1 adjusted for age, sex, and country of birth. ^b^Model 2 adjusted for age, sex, country of birth, and epigenetic age; ^c^Model 3 adjusted for age, sex, country of birth, and SRH.

Table S3: The association between SRH and mortality adjusted for more covariates (*n*=802, *n*_deaths_=250).

| **Characteristics** | ***GrimAge*** | | | ***PhenoAge*** | | | ***DunedinPACE*** | | |
| --- | --- | --- | --- | --- | --- | --- | --- | --- | --- |
|  | **HR** | **95% CI** | ***P*-value** | **HR** | **95% CI** | ***P*-value** | **HR** | **95% CI** | ***P*-value** |
| SRH | 1.28 | 1.09, 1.50 | 0.003 | 1.28 | 1.09, 1.51 | 0.003 | 1.30 | 1.11, 1.52 | 0.001 |
| Epigenetic age | 1.42 | 1.22, 1.66 | 9×10^-6^ | 1.15 | 1.02, 1.30 | 0.02 | 1.26 | 1.10, 1.44 | 9×10^-4^ |
| Sex |  |  |  |  |  |  |  |  |  |
| Female | Reference group | | | | | | | | |
| Male | 0.64 | 0.41, 1.00 | 0.05 | 0.76 | 0.49, 1.18 | 0.22 | 0.74 | 0.48, 1.15 | 0.19 |
| Country of birth |  |  |  |  |  |  |  |  |  |
| Australia/New Zealand/Other | Reference group | | | | | | | | |
| Greece | 0.79 | 0.29, 2.18 | 0.65 | 0.78 | 0.28, 2.18 | 0.64 | 0.71 | 0.26, 1.97 | 0.51 |
| Italy | 0.65 | 0.38, 1.13 | 0.13 | 0.71 | 0.41, 1.22 | 0.22 | 0.67 | 0.39, 1.16 | 0.15 |
| UK/Malta | 0.85 | 0.55, 1.33 | 0.48 | 0.85 | 0.55, 1.33 | 0.48 | 0.84 | 0.54, 1.30 | 0.43 |
| Age (years) | 1.13 | 1.10, 1.16 | <2×10^-16^ | 1.13 | 1.10, 1.16 | <2×10^-16^ | 1.13 | 1.10, 1.16 | <2×10^-16^ |
| Soma-6 | 0.98 | 0.92, 1.05 | 0.59 | 0.98 | 0.92, 1.05 | 0.56 | 0.97 | 0.92, 1.04 | 0.41 |
| Smoking status |  |  |  |  |  |  |  |  |  |
| Never smokers | Reference group | | | | | | | | |
| Current smokers | 1.09 | 0.52, 2.28 | 0.82 | 2.15 | 1.11, 4.16 | 0.02 | 1.84 | 0.94, 3.60 | 0.07 |
| Former smokers | 0.91 | 0.68, 1.23 | 0.53 | 1.14 | 0.87, 1.50 | 0.34 | 1.15 | 0.88, 1.50 | 0.32 |
| BMI | 0.65 | 0.47, 0.89 | 0.01 | 0.63 | 0.46, 0.87 | 0.005 | 0.63 | 0.46, 0.87 | 0.005 |
| Waist circumference | 1.69 | 1.22, 2.34 | 0.002 | 1.74 | 1.25, 2.41 | 0.001 | 1.69 | 1.22, 2.35 | 0.002 |
| Height | 1.05 | 0.85, 1.29 | 0.67 | 1.04 | 0.84, 1.29 | 0.70 | 1.06 | 0.86, 1.30 | 0.61 |
| Blood glucose concentration | 1.24 | 1.10, 1.41 | 7×10^-4^ | 1.23 | 1.09, 1.40 | 9×10^-4^ | 1.23 | 1.09, 1.40 | 0.001 |
| LDL cholesterol concentration | 0.91 | 0.79, 1.05 | 0.18 | 0.91 | 0.79, 1.05 | 0.18 | 0.91 | 0.79, 1.04 | 0.17 |
| HDL cholesterol concentration | 0.98 | 0.85, 1.12 | 0.73 | 0.98 | 0.85, 1.12 | 0.74 | 1.00 | 0.87, 1.15 | 0.98 |
| Systolic blood pressure | 1.14 | 0.99, 1.31 | 0.06 | 1.16 | 1.01, 1.33 | 0.03 | 1.14 | 0.99, 1.31 | 0.06 |
| Resting heart rate | 1.07 | 0.95, 1.22 | 0.26 | 1.11 | 0.98, 1.25 | 0.11 | 1.09 | 0.96, 1.23 | 0.17 |

*The model adjusted for age, sex, country of birth, fatigue, waist circumference, blood glucose level, total cholesterol level, systolic blood pressure, rest heart rate, and epigenetic age. Epigenetic age, waist circumference, blood glucose concentration, LDL cholesterol concentration, HDL cholesterol concentration, systolic blood pressure, and resting heart rate were standardised to a mean of 0 and standard deviation of 1.

Table S4: The C-index for each model

| Models | C-index | |
| --- | --- | --- |
|  | Categorical SRH | Continuous SRH |
| *GrimAge* | 0.590 | - |
| *PhenoAge* | 0.552 | - |
| *DunedinPACE* | 0.574 | - |
| SRH | 0.589 | 0.589 |
| Model 0 (age+sex+country of birth) | 0.748 | - |
| Model 1 (Model 0+SRH) | 0.757 | 0.756 |
| Model 0+ *GrimAge* | 0.763 | - |
| Model 0 + *PhenoAge* | 0.754 | - |
| Model 0 + *DunedinPACE* | 0.760 | - |
| Model 2 (Model 1 + *GrimAge*) | 0.769 | 0.768 |
| Model 2 (Model 1 + *PhenoAge*) | 0.761 | 0.761 |
| Model 2 (Model 1 + *DunedinPACE*) | 0.764 | 0.764 |
| Model 3 (Model 1+*GrimAge*+*PhenoAge*+*DunedinPACE*) | 0.770 | 0.769 |
| Model 2+SRH**GrimAge* | - | 0.770 |
| Model 2+SRH**PhenoAge* | - | 0.762 |
| Model 2+SRH**DunedinPACE* | - | 0.765 |
| Model 1+*GrimAge*+risk factors | - | 0.782 |
| Model 1+*PhenoAge*+risk factors | - | 0.777 |
| Model 1+*DunedinPACE*+risk factors | - | 0.777 |

^a^Risk factors including fatigue (soma-6 score), waist circumference, smoking status, blood glucose level, HDL and LDL cholesterol concentrations, systolic blood pressure, and resting heart rate.

All epigenetic ageing measures are age-adjusted.
